# Interpretable Biomechanical Feature Selection for VR Exercise Assessment Using SHAP and LDA

**DOI:** 10.3390/s26020464

**Published:** 2026-01-10

**Authors:** Urszula Czajkowska, Magdalena Żuk, Michał Popek, Celina Pezowicz

**Affiliations:** Faculty of Mechanical Engineering, Wroclaw University of Science and Technology, Wybrzeże Wyspianskiego 27, 50-370 Wrocław, Poland; magdalena.zuk@pwr.edu.pl (M.Ż.); m.popek.wroc@gmail.com (M.P.)

**Keywords:** virtual reality (VR), human movement quality assessment (HMQA), biomechanics, motion analysis, linear discriminant analysis (LDA), SHapley additive explanations (SHAP)

## Abstract

**Highlights:**

**What are the main findings?**

- Both LDA and SHAP consistently selected overlapping key biomechanical features, confirming high agreement in feature relevance.
- Angular features achieved higher classification effectiveness (F1 = 0.89), whereas distance-based features provided greater stability and resistance to calibration inaccuracies.

**What are the implications of the main findings?**

- The identified angular and distance-based features provide a starting point for developing more advanced models, which should deliver even better performance when applied to richer algorithms and expanded datasets.
- Reliable assessment of lateral lunges in VR can be achieved with a simplified sensor set: headset plus sensors on feet and shanks.

**Abstract:**

Virtual reality (VR) technologies are increasingly applied in rehabilitation, offering interactive physical and spatial exercises. A major challenge remains the objective assessment of human movement quality (HMQA). This study aimed to identify biomechanical features differentiating correct and incorrect execution of a lateral lunge and to determine the minimal number of sensors required for reliable VR-based motion analysis, prioritising interpretability. Thirty-two healthy adults (mean age: 26.4 ± 8.5 years) performed 211 repetitions recorded with the HTC Vive Tracker system (7 sensors + headset). Repetitions were classified by a physiotherapist using video observation and predefined criteria. The analysis included joint angles, angular velocities and accelerations, and Euclidean distances between 28 sensor pairs, evaluated with Linear Discriminant Analysis (LDA) and SHapley Additive exPlanations (SHAP). Angular features achieved higher LDA performance (F1 = 0.89) than distance-based features (F1 = 0.78), which proved more stable and less sensitive to calibration errors. Comparison of SHAP and LDA showed high agreement in identifying key features, including hip flexion, knee rotation acceleration, and spatial relations between headset and foot or shank sensors. The findings indicate that simplified sensor configurations may provide reliable diagnostic information, highlighting opportunities for interpretable VR-based rehabilitation systems in home and clinical settings.

## 1. Introduction

The rapid development and growing availability of virtual reality (VR) technology mean that it is being applied increasingly across a wide range of scientific disciplines. Helou et al. provide a comprehensive overview of VR applications in medicine [1], highlighting areas such as rehabilitation, public health training, clinical training for professionals, and medical image visualisation. VR supports rehabilitation by serving as a multifaceted tool that offers interactive physical and spatial exercises. Examples include its use in the rehabilitation of neurological patients [2,3] and orthopaedic patients [4,5]. VR can also be used to analyse the movements of athletes or patients, enabling researchers to better understand the mechanics of movement sequences and to develop personalised training sequences that enhance performance or support rehabilitation [6,7,8].

The most commonly used VR systems consist of base stations, goggles (headsets), and handheld controllers. These kits enable the mapping of the user’s body and objects within a virtual environment, while also providing natural and intuitive interaction with its elements. The basic equipment can be complemented by HTC Vive Tracker sensors, which operate with headsets and controllers in a VR environment, but can also function independently as a standalone motion-tracking system. These sensors enable the recording of quantitative data describing performed motor tasks, such as joint angles and body-segment velocities and positions [5,6,7]. This VR accessory-based tracking system provides a cost-effective solution for recording motion in six degrees of freedom (6DOF) within a global coordinate system, allowing for the measurement of both the position and orientation of objects. Numerous studies highlight the potential of this technology for analysing the movements of VR users, demonstrating the high precision of the data obtained and the repeatability of results comparable to those of advanced optoelectronic systems [9,10,11,12].

As part of the tracking system, a proprietary eMotion application has been developed that serves as a platform for the acquisition and analysis of kinematic data (Poland, Wroclaw). The programme enables the recording and processing of motion data from VR devices, as well as the calculation of lower-limb joint angles using two implemented measurement protocols: Simplified6DOF, which allows for rapid measurements without anatomical calibration, and ISB6DOF, which is compliant with the guidelines of the International Society of Biomechanics and requires calibration. A detailed description of the application is provided by Żuk et al. [9].

In parallel with the tracking system, a VR motion-based game was developed to support the rehabilitation and training process. This objective is achieved through engaging VR gameplay, during which users are encouraged to perform planned movement sequences; points are earned by hitting or avoiding virtual objects, whether stationary or moving. An example of its application is the use of the system to conduct exercise sessions for orthopaedic patients [4].

However, designing the game mechanics and creating a motion-tracking system are not sufficient to achieve the broader goal of assessing the quality of the repetitions performed, as users can collect points in various ways rather than through a single, strictly planned movement pattern.

Human Motion Quality Assessment (HMQA) refers to the objective evaluation of the quality and efficiency of human movements during physical activity, training, or rehabilitation. Its purpose is both to monitor the correctness of performed motor tasks and to identify potential errors or irregularities in movement. HMQA can be understood in a broad context, ranging from comparisons of movement repeatability with expert reference patterns to assessments of patients’ movements against normative data or those of healthy individuals [13,14]. In practice, motion analysis involves the measurement of kinematic parameters (joint angles, body-segment trajectories, velocities, and accelerations), spatiotemporal parameters (e.g., for gait: speed, cadence, stride length and width, gait-phase duration, symmetry, and variability), and selected structural and functional indicators (e.g., inter-joint distances, segment rotation) [15,16,17,18,19,20,21]. Movement quality assessment can be performed using rules, templates, or statistical methods, allowing for the classification of movements, the identification of errors, and the monitoring of exercise repeatability. HMQA can also be considered a specific form of *human activity recognition (HAR)*, in which movement classification is based on subtle differences in the performance of motor tasks [22,23].

Human motion analysis commonly uses basic preprocessing procedures, such as filtering, segmentation, and normalisation [9,24]. In contrast, classical techniques used in the main analysis include signal transforms (FFT, STFT, Wavelet) [25,26,27,28], Dynamic Time Warping (DTW) [29,30], Empirical Mode Decomposition (EMD) [31], Kalman filters [32,33], and probabilistic and statistical models, including Hidden Markov Models (HMM) [34,35,36], Dynamic Bayesian Networks [37,38], Gaussian Mixture Models (GMM) [39,40], and ARMA [41] and ARIMA [42] time-series models. Contemporary approaches also include machine-learning and deep-learning algorithms such as SVM [43,44], KNN [43,44,45], PCA [46,47], LDA [48], multiple regression [49], and cluster analysis [50] as well as ANN [51,52], LSTM [53,54], BiLSTM [55,56], CNN, CNN-LSTM, FCN [57,58,59,60], ResNet [61], and transformer-based models [62,63].

The number of possible methods, models, and parameters is vast, and many advanced algorithms remain a ‘black box’ in practice, making it difficult to interpret the results and determine which features are responsible for correct classification and why. One solution is to use SHapley Additive exPlanations (SHAP), which provide a better understanding of the contribution of individual features to the decisions made by the classification model [64].

The aim of this study is to identify the key biomechanical features that distinguish between correct and incorrect performance of a selected physical exercise, and to determine the minimum number of sensors required for reliable analysis of VR users’ movement data. The priority was not to maximise classification accuracy, but to employ methods that ensure high interpretability of the results.

## 2. Materials and Methods

### 2.1. Measurement Setup and Procedure

Thirty-two healthy, injury-free adults (13 women and 19 men) were included in the study (mean age: 26.4 ± 8.5 years; mean height: 176 ± 9.1 cm; mean mass: 69.8 ± 10.6 kg). The study was approved by the local Ethics Committee, and all participants provided written informed consent.

Seven HTC Vive Trackers (versions 2.0 and 3.0) and an HTC Vive head-mounted display were used to record lower-limb joint kinematics, together with two Lighthouse 2.0 base stations from the HTC Vive Pro Full Kit (HTC, New Taipei City, Taiwan), placed 5.7 m apart. All sensors were connected to a ROG Strix G15 laptop (Asus, Taipei, Taiwan) using an 8-port powered USB 3.0 hub (iTec, Ostrava, Czech Republic).

Participants were prepared for data collection by attaching Vive Trackers to their lower limbs using Vortex attachment straps. The sensors were placed on the following locations: the right side of the pelvis, the lateral aspects of the thighs, the distal lateral shanks, and the dorsal aspects of the feet. In addition, participants wore the VR headset for tracking purposes only, without visual occlusion, and held the controllers in their hands.

Measurements were performed using the acquisition module of the proprietary eMotion software (version 2.8), which collects data from Vive Trackers and the HTC Vive VR system, as described in detail by Żuk et al. [9]. The study used the Simplified 6DOF protocol, in which joint angles are defined as angles between trackers; therefore, precise sensor placement along the axes of the body segments is crucial for measurement accuracy. The protocol assumes that the axes of the body segments coincide with the axes of the trackers. Only the position of the tracker on the foot was not significant, as a correction procedure was applied whereby an ankle joint angle of 0° was assumed in an upright standing position with parallel feet. Data were recorded during the exercises and subsequently processed in the eMotion software to calculate joint angles. During data acquisition with the Vive Trackers, video recordings were captured simultaneously using a Galaxy S20 FE smartphone (Samsung Electronics, Suwon, South Korea), which recorded at a resolution of 1920 × 1080 pixels and 30 frames per second.

**Figure 1:**
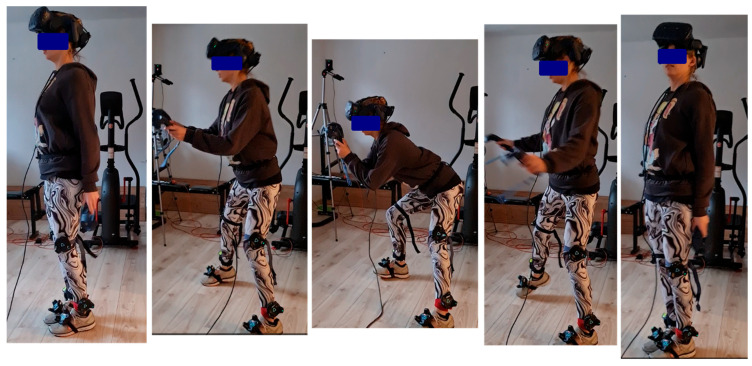
Correct execution of the lateral lunge exercise.

**Figure 2:**
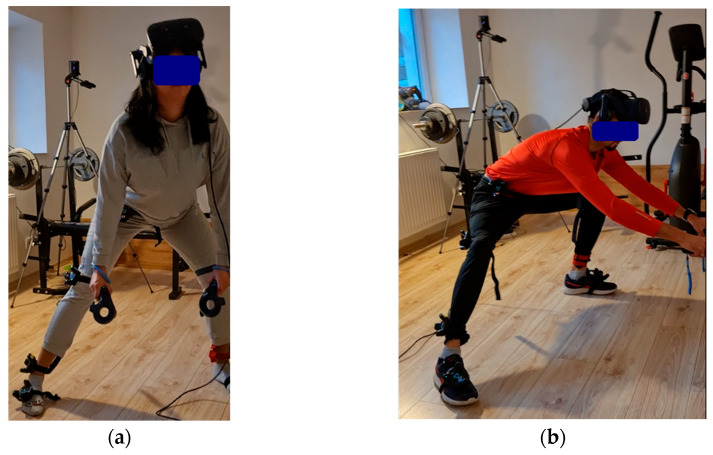
Incorrect execution of the lateral lunge: (**a**) absence of pelvic lowering; (**b**) flexion of both lower limbs accompanied by a hunched posture.

After recording a static standing position, participants received instructions and performed a side lunge, completing three repetitions on each side. If additional repetitions were performed, all of them were included in the analysis. In three cases, the measurement was repeated twice. A total of 211 lunge repetitions were recorded, of which 110 were classified as correct and 100 as incorrect. The assessments and category assignments were performed by a physiotherapist according to predefined criteria based on the observation and analysis of the video recordings. The successive phases of the exercise performed correctly, along with examples of common errors, are shown in Figure 1 and Figure 2.

The starting position for the exercise involved standing with the feet parallel and no more than hip-width apart, with the torso upright and the pelvis in a neutral position. The leading leg then moved laterally in a straight line, without rotation or forward displacement, while the knee remains aligned above the ankle as the centre of gravity is lowered. The hip was lowered with a simultaneous backward movement of the pelvis. During this movement, the supporting leg remained straight or slightly flexed, with the foot firmly grounded. The torso leaned slightly forward while maintaining the natural spinal curvature, without slouching, and the pelvis remained symmetrical without rotation. The range of motion resulted from hip and knee flexion and was smooth and within physiological limits. The return to the starting position was achieved by dynamically pushing off with the leading leg, while maintaining balance and active control of the torso.

Repetitions that failed to meet the above criteria were considered incorrect. The most common errors observed included excessive forward displacement of the leading knee during lowering, simultaneous flexion of both lower limbs instead of only the leading leg, failure to clearly lower the centre of gravity, and performing the movement without fully returning to the starting position (defined as placing the feet hip-width apart and keeping the torso upright).

### 2.2. Preprocessing

Data analysis was performed in the MATLAB environment (R2026b). Data were prepared in several stages, including the processing of sensor trajectories and joint angles. After manual segmentation into individual repetitions, the signals were filtered using a 6 Hz Butterworth filter, normalised to 101 frames, and corrected for offsets introduced by the eMotion angle calculation algorithms. The correction involved calculating the mean of the first five frames and shifting the signal by 180° if the absolute value of the mean exceeded 165°. Sign corrections were also applied to selected hip, knee, and ankle rotations (to account for differences in coordinate systems), in accordance with the system developers’ recommendations to standardise the directions of rotation [9]. The limbs were treated functionally (leading vs. supporting leg).

During this stage, the quality of the raw sensor trajectories was checked, and unnatural signal jumps or losses were identified. The affected fragments were marked as NaN in both the trajectories and the corresponding angles, which avoided the removal of entire observations and preserved as many samples as possible. At this stage, the following signals were identified as defective: 65 from the hip sensor, 20 from the right thigh, 12 from the right foot, 8 from the left thigh, 11 from the left shank, and 7 from the left foot. Subsequently, a visual inspection of the angular waveforms was performed. In the case of further anomalies, only the angular data were selectively marked as NaN. The analysis also identified artefacts arising from the limitations of representing rotation using Euler angles (including gimbal lock). These artefacts caused, among other effects, flattened knee flexion waveforms with simultaneous unnatural variability in the abduction angle; in such cases, the affected data were also rejected. The final results of the angular data cleaning process are summarised in the following Table 1.

**Figure 3:**
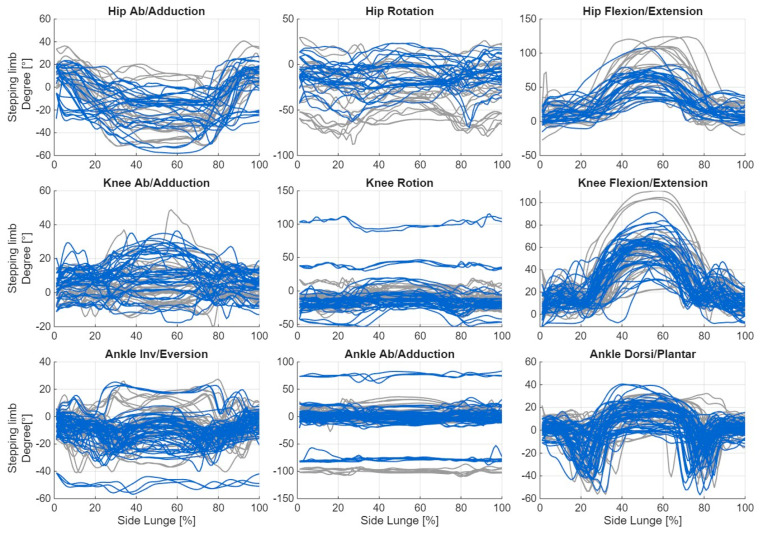
Plot of changes in joint angle values during the execution of the lateral lunge for the stepping limb, shown separately for consecutive repetitions (blue: incorrect, grey: correct).

**Figure 4:**
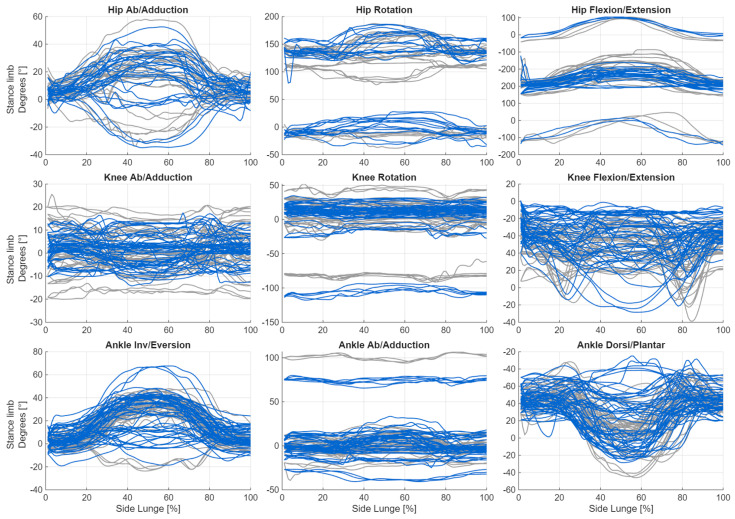
Plot of changes in joint angle values during the execution of the lateral lunge for stance limb, shown separately for consecutive repetitions (blue: incorrect, grey: correct).

Below are graphs showing changes in joint angles for movements performed on the right side. Despite the applied correction, visible offsets remain in the data. It is also noteworthy that the directions of the curves are not always consistent (e.g., abduction/adduction of the hip joint in the supporting leg). The next step was to compute angular velocities and accelerations which, as derivatives of the signal, are independent of both the offset and the curve direction. These derived measures can provide valuable information about the movement strategy, even when the direction of rotation is uncertain. Figure 3 and Figure 4 illustrate the changes in joint angle values during the execution of the lateral lunge, presented separately for consecutive repetitions.

Since the trajectories themselves depend on the coordinate system as well as on individual anthropometric characteristics, it was decided to analyse the distances between the sensors. These were calculated as the Euclidean distances between all possible pairs of sensors at each time instant, according to the formula:

(1) $$d=\sqrt{(x_{A}-x_{B}{)}^{2}+(y_{A}-y_{B}{)}^{2}+(z_{A}-z_{B}{)}^{2}},$$

**Figure 5:**
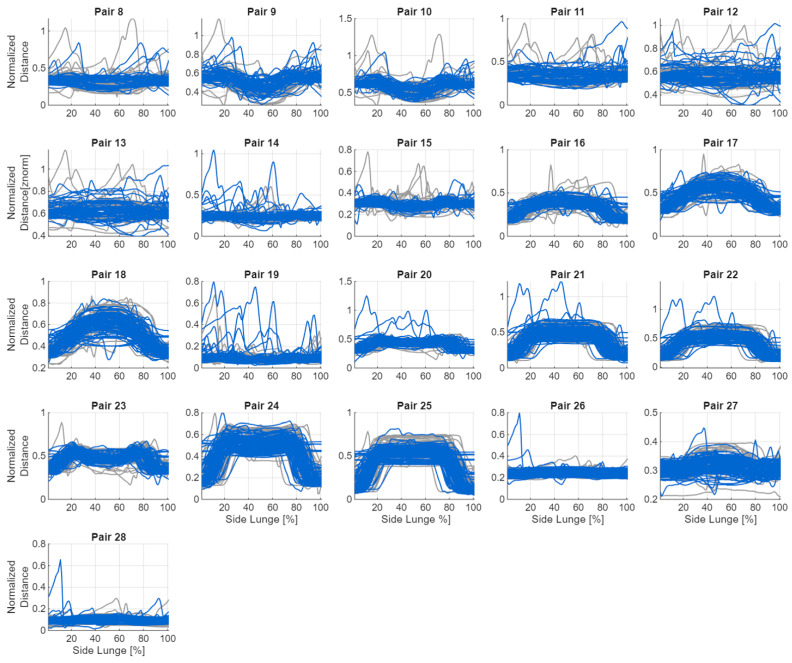
Changes in distances between sensor pairs without goggles during the exercise (blue—incorrect, grey—correct).

**Figure 6:**
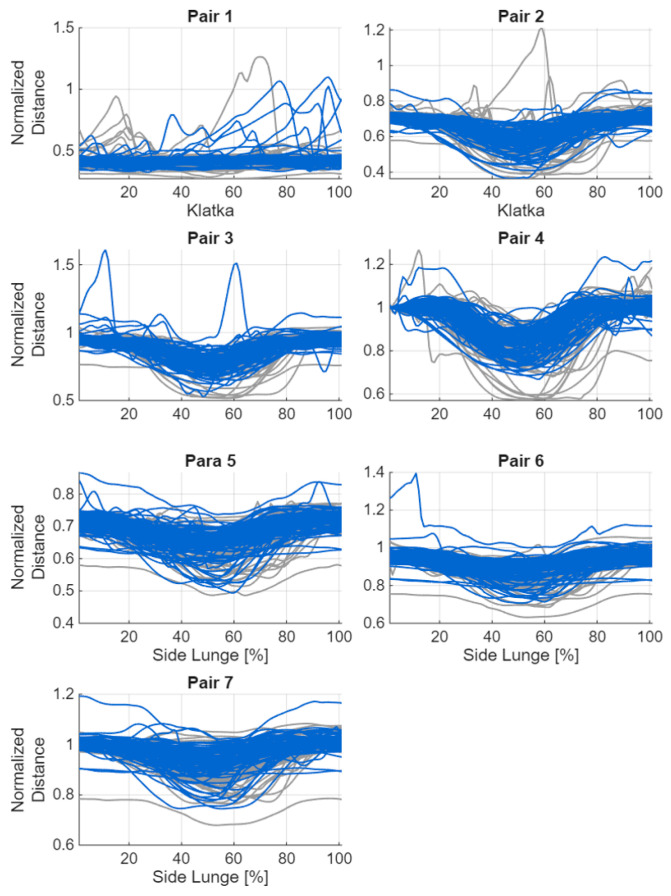
Changes in distances between sensor pairs with goggles during the exercise (blue: incorrect, grey: correct).

If the coordinates of any of the sensors contained a NaN value, a missing value was assigned to the corresponding sensor pair and frame. To limit the impact of anthropometric differences, all distances were normalised relative to a reference distance—the distance between the goggles and the foot of the leading limb in the first frame. When the coordinates of this foot contained NaN, the distance between the goggles and the supporting foot was used instead. All spatial values were then divided by this reference distance to obtain a comparable scale across participants. All 28 possible sensor pairs were analysed, including the seven trackers on the lower limbs and the goggles (Table 2). Hand trajectories were excluded because the subjects did not receive clear instructions on upper-limb positioning. Figure 5 and Figure 6 show graphs of the changes in distance between the sensors during the exercise.

The procedure described above ensured consistent, orderly, and standardised datasets covering joint angles with their corresponding angular velocities and accelerations, as well as the distances between the sensors used during the measurements. Each repetition was also labelled with its correctness (‘correct’/‘incorrect’), the side on which the exercise was performed (‘right’/‘left’), and a ‘subjectID’ to identify repetitions performed by the same participant. The data prepared in this way provided the foundation for the subsequent analysis.

### 2.3. Statistical Analysis

Due to frequent shifts in the angular signals relative to the OY axis, only offset-insensitive measures were calculated for the angles, namely standard deviation, range of motion (ROM), skewness, and kurtosis. For angular velocities and accelerations, a more complete set of statistics was computed, including the mean, median, minimum, maximum, standard deviation, quartiles, coefficient of variation (CV), ROM, skewness, and kurtosis. The same set of measures was calculated for the distances between sensors. In total, 72 angular features, 200 velocity and acceleration features, and 224 distance-based features were obtained.

The analysis was conducted at the level of individual repetitions, comparing correct and incorrect performances. The normality of each distribution was assessed using the Shapiro–Wilk test, followed by either Student’s *t*-test or the Wilcoxon–Mann–Whitney test. For each feature, the *p*-value and effect size (Cohen’s d) were calculated. In parallel, a correlation analysis (Spearman) was performed, and redundant features were removed (|r| > 0.98).

**Figure 7:**
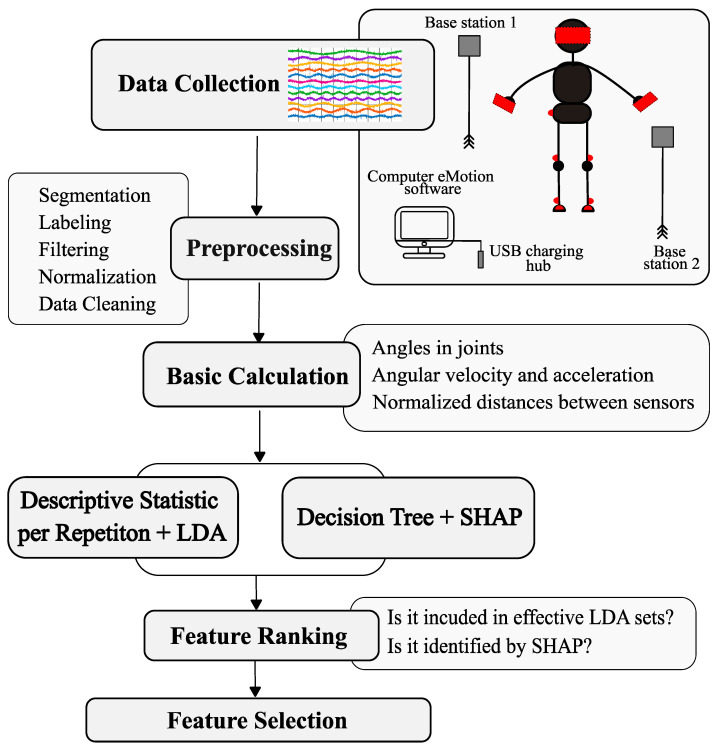
Workflow of the analysis procedure applied in the study. Red color—schematic marking of sensors during measurements.

In addition, the classification potential of each feature was assessed using the area under the curve (AUC) of the receiver operating characteristic (ROC), identifying variables that simultaneously met the following criteria: AUC ≥ 0.55, Cohen’s d ≥ 0.5, and *p* < 0.1. This method identified seven distance features and 117 angular features. Due to the small number of distance features meeting the AUC criterion, feature sets selected on the basis of significance and correlation tests (48 features) were used for further analysis. The final set consisted of statistical parameters derived from angular and distance signals (e.g., ROM, deviations, quartiles, and distribution shape measures), which served as input data for the LDA classification model. Figure 7 illustrates the workflow of the study.

### 2.4. Linear Discriminant Analysis (LDA)

To determine which movement parameters—both individual features and combinations thereof—best distinguished between correct and incorrect performances, classical linear discriminant analysis was applied (Statistics and Machine Learning Toolbox; fitcdiscr function with equal a priori class probabilities: ‘Prior’, ‘uniform’). This method identifies a linear boundary between groups based on the feature distributions, assuming a common covariance matrix [65].

To prevent information leakage between the training and test sets, the data were split according to participant ID so that repetitions from the same individual never appeared in both sets simultaneously. Each experiment was repeated twenty times, with participants randomly assigned to five validation folds per iteration. In each iteration, the training data were standardised using a Z-score, while the test data were rescaled using the mean and standard deviation parameters calculated from the training set. This approach ensured standardisation of the feature scale while maintaining the isolation of the test data. The results were then averaged, allowing assessment of both the effectiveness and repeatability of the models. The significance of the results was verified using a simplified permutation test (50 permutations) without group validation.

The features used were statistical parameters computed for individual repetitions (e.g., range of motion of a given joint, standard deviation, median, or kurtosis), with each feature representing a single numerical value characterising the course of the movement.

The analysis compared sets of features of varying sizes: single features, pairs, triples, and sets of four (the latter were obtained by adding one feature to the ten best sets from the previous step). The number of features was limited to a maximum of four in accordance with recommendations for the stability of linear classifiers, which state that the number of samples in each class should be at least 5–10 times greater than the number of parameters [66]. This condition was satisfied in both classes.

The effectiveness of the models was assessed using four metrics: accuracy, F1 for the incorrect class, AUC, and standard deviation of F1 (Std F1). This set of metrics allowed for simultaneous evaluation of the accuracy, stability, and reliability of the classification. Particular emphasis was placed on the F1 measure for the incorrect class, as in the context of the designed application it is especially important to correctly detect incorrect performances, which are most relevant for the safety of training or rehabilitation. Table 3 presents the best combinations of trajectory features, while Table 4 shows the best combinations of angular features, highlighting the biomechanical parameters that most effectively distinguished between correct and incorrect performances. The F1-score, which combines precision and sensitivity, was used to evaluate classification quality, reflecting the model’s effectiveness in recognising incorrect exercise executions.

### 2.5. SHapley Additive Explanation (SHAP)

The classification analysis considered all calculated statistical features—both those selected in previous significance tests and other descriptive parameters. The features were grouped into blocks, which in practice represented an equal division of the full feature list according to their order. This approach facilitated the organisation of the data and the interpretation of the impact of individual feature groups on the model, without omitting any features. The detailed composition of the blocks is not presented; however, each block included a complete set of features for all signal types.

The analysis was performed in the MATLAB environment (Statistics and Machine Learning Toolbox). For each block, twenty cross-validation iterations were conducted using a 5-fold scheme in two variants: with grouping by participants and with random assignment. The training data were standardised separately in each iteration using Z-scores, while the test data were scaled using parameters derived from the training set. A single decision tree (fitctree) was used for classification. The impact of individual features was assessed based on Shapley values, calculated using the shapley function, with the global importance of each feature defined as the average absolute value of its Shapley coefficients. When Shapley values were unavailable, the predictorImportance measure was used as an alternative. The analysis was performed only for blocks that met the effectiveness criteria (presented in Table 5), and feature rankings were generated for these blocks (examples are listed in Table 6).

Selected features from the blocks were combined into a new data matrix, on which cross-validation was performed again and SHAP values were calculated, generating a ranking of features according to their average impact. In the next iteration, 50% of the highest-ranked features were retained to assess the effect of feature reduction on model performance. For angular data, only one block met the criteria in grouped validation (acc = 0.60; F1 = 0.61), with half of its features advancing to subsequent stages. In random validation, four blocks were fully included and ten blocks partially included in the subsequent stages. However, the metrics were highly variable.

In the case of angular features, because the results deteriorated after combining selected feature sets and recalculating the metrics, only the features from the best-rated random-validation blocks and from the best grouped-validation block were retained for further analysis, provided that their weights were greater than zero. This procedure resulted in a total of 42 features. A summary of selected parameters is provided in Table 7. Notably, the selected angular features mainly relate to the hip joint of the supporting leg, whereas for velocity and acceleration, features associated with the hip and knee joints of the leading leg predominate.

For the distance data, the same feature selection procedure was applied as for the angular data. In the second stage, a six-step SHAP-based selection was carried out, with the number of analysed features reduced by half at each step. In the case of random validation, the best results were achieved using an eleven-element feature set (details are provided in Table 8). However, a larger 41-element set was ultimately selected for constructing the rankings, as it provided both good performance and the highest stability, and its size was comparable to the angular feature set. For grouped validation, none of the blocks met the criteria required for inclusion in the subsequent stages of analysis.

**Figure 8:**
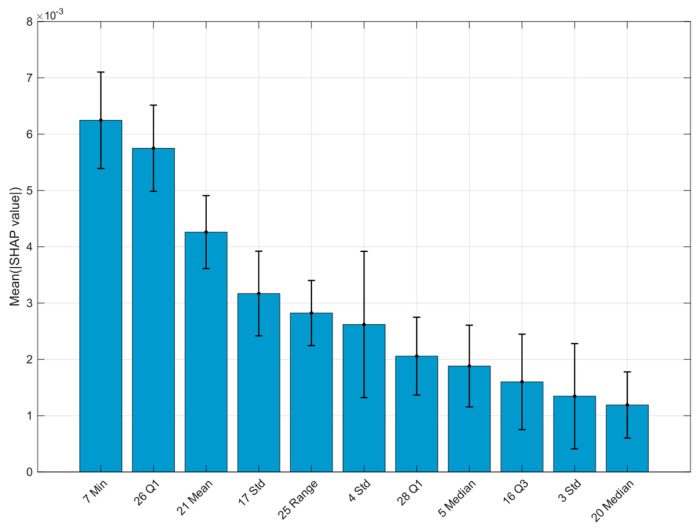
SHAP-based ranking of distance features. Pair numbers and statistical descriptors are shown on the x-axis.

As an illustration of the approach used, a SHAP bar chart (feature importance plot) is presented for the block of distance features with the highest classification effectiveness, showing the global importance of the individual parameters (Figure 8). No analogous visualisations are provided for the remaining blocks, as they do not contribute any additional key information within the adopted framework.

### 2.6. Feature Selection

The next stage involved a ranking analysis of the features selected using two independent approaches, with the aim of identifying biomechanical variables consistently regarded as significant. Features selected by SHAP for all data types (angles, velocities, accelerations, distances) and those highlighted by LDA were considered, but only from effective sets (F1 ≥ 0.70, AUC ≥ 0.75), These sets were divided into individual features, and their frequency of occurrence was counted. The analysis then focused on identifying features that recurred most often in the most significant sets. Features that recurred multiple times and had the greatest impact on model performance were considered the most important, allowing a comparison of the relative importance of individual variables and identification of key parameters for classification.

## 3. Results

In the angular-data analysis, 4412 of the 13,440 LDA sets met the predefined criteria. These sets contained 115 unique features, including 17 that overlapped with features selected using SHAP. Across the complete set of angular features considered, the value of joint angles appeared in the effective LDA sets 15 times (Table 9).

The features appearing most frequently in the effective LDA sets were also selected by SHAP. All features related to joint angle values and identified by both LDA and SHAP corresponded to hip flexion in the supporting leg. The effective LDA sets included 52 features for angular velocities and 48 features for angular accelerations. These findings suggest that movement dynamics is an important indicator. They also indicate that correcting for offsets when computing first- and second-order derivatives from angle values may be important.

Table 10 summarises the angular features that occur most frequently in the effective LDA sets, as well as those identified by SHAP.

In the case of distance data and LDA, 36 of the 3926 evaluated sets met the predefined effectiveness thresholds. These were constructed from 14 unique features tested in multiple combinations, including three that overlapped with features identified by SHAP. These three highlighted features correspond to the following sensor pairs:

Goggles—foot of the leading leg (100%)

Shank of the leading leg—shank of the supporting leg (11.1%)

Shank of the leading leg—foot of the leading leg (8.3%)

Table 11 summarises the results for the distance features.

In addition, it is worth noting features that recur frequently in effective LDA sets, even though they were not identified in the SHAP analysis. These are:

Standard deviation of the distance between the goggles and the thigh of the leading leg.

Median distance between the shank of the leading leg and the foot of the supporting leg.

Standard deviation of the distance between the shank of the leading leg and the foot of the supporting leg.

Minimum acceleration in the hip joint of the supporting leg in rotation and flexion/extension.

The analysis showed that key diagnostic information can be obtained primarily from the goggles and sensors placed on the feet and shanks for distance analysis, and from the hip and knee joint angles for angular data.

## 4. Discussion

To achieve the study objective, the side lunge was selected as the motor task due to its widespread use in functional training and lower-limb rehabilitation [67]. This exercise involves a complex, multi-joint movement in an open kinematic chain, producing rich and diverse biomechanical signals. An additional advantage is its asymmetrical nature, which allows assessment of both the supporting and stepping limbs, as well as analysis of how sensor placement affects data quality. Consequently, the side lunge is a sufficiently challenging yet controlled task, enabling reliable evaluation of both the significance of individual movement characteristics and sensor configurations in VR conditions.

The variety of available approaches in human motion analysis reflects the complexity of the field and makes it challenging to select appropriate methods for implementation and interpretation in practical systems, such as VR applications supporting rehabilitation. This paper therefore focuses on identifying the key biomechanical features that distinguish between correct and incorrect lunge execution, and on determining the number of sensors required for reliable analysis of VR users’ movement data—a first step toward developing simple yet effective methods for assessing movement in a VR environment. To this end, simple descriptive statistical parameters were analysed, and methods with high interpretability were applied, namely LDA and a decision tree model interpreted using SHAP.

These methods have previously been applied in similar contexts. For example, SHAP has been used for feature selection in classifying types of locomotion transitions, demonstrating that this technique can significantly reduce the number of channels analysed while maintaining high classification accuracy [22,68]. LDA has also been employed in motion analysis, including the classification of fitness exercises using data from inertial sensors [48], and in combination with HMM for feature reduction in HAR tasks, showing that this approach can substantially improve balanced classification accuracy [69].

The integration of SHAP provides a critical advantage over ‘black-box’ deep learning models, such as the LSTM or CNN architectures used by Spilz and Munz [53]. While deep learning often yields high accuracy, it lacks clinical interpretability. By using SHAP, we can pinpoint specific biomechanical indicators, such as knee rotation acceleration, that contribute to the classification, allowing physiotherapists to provide targeted, evidence-based feedback to patients.

Based on the analysis, six features were selected that were jointly identified by the linear LDA model and the non-linear decision tree model, along with four additional features that were not selected by SHAP but appeared frequently in effective LDA sets. These features were considered to have the greatest potential for assessing lunge performance and, importantly, are supported by a clear biomechanical rationale (Table 12).

The analysis revealed that key information for distinguishing between correct and incorrect lunge executions can originate from different sets of sensors, depending on the type of data analysed. For distance-related parameters, the most informative signals came from the goggles and sensors placed on the feet and shanks, whereas in the analysis of angular data, features related to the hip and knee joints predominated. Importantly, among the angular features, angular acceleration of knee joint rotation of the leading leg emerged most frequently, highlighting the potential value of derivatives, rather than just the raw joint angles. It is worth noting that the range of motion in this plane, with the joint flexed, can reach up to 44° [70]. At the same time, all joint-angle features selected by both methods simultaneously referred to hip flexion and extension in the supporting leg.

Clear signal offsets were observed in the angular data, likely resulting from calibration imperfections, measurement errors, or variability in sensor placement during recordings. This phenomenon should be considered natural, particularly in the context of home use, where VR users are unlikely to perform calibration as precisely as in laboratory conditions. Consequently, it is especially important to design analysis methods that are robust to baseline shifts and uncertainty in signal orientation.

The LDA classification results indicated that angular data outperformed distance data (F1 = 0.89 ± 0.007 vs. 0.78 ± 0.004 for the best sets), although angular features were more sensitive to local signal fluctuations and incurred a higher computational cost. In contrast, distance features were more stable, less computationally demanding, and more resistant to calibration imperfections. Our classification performance (F1 = 0.89 for angular data) is consistent with other state-of-the-art systems for human movement quality assessment (HMQA),. For instance, Yu and Xiong achieved a correlation of r = 0.86 using DTW for rehabilitation exercises [29], while Pereira et al. reported a 96% accuracy in error detection using pure inertial sensors [71]. However, our findings align with Vox et al. regarding the inherent limitations of VR-based tracking, where joint angle discrepancies can range from ±6° to ±42° depending on the sensor configuration and calibration [72].

Practical implementation in home settings faces significant hardware challenges. Our study confirmed that thigh-mounted sensors are prone to shifting during dynamic lunges, and the hip sensor frequently suffers from loss of line-of-sight with the base stations. These real-world constraints justify our focus on a simplified sensor configuration (headset, shanks, and feet), which proved more robust and less sensitive to such artefacts while maintaining diagnostic reliability. Consequently, the choice of appropriate sensors and features should depend on the target application. In home systems, where simplicity and robustness to user errors are priorities, distance-based metrics represent a promising solution. In contrast, in clinical contexts, where measurement conditions and sensor placement can be controlled more precisely, full kinematic analysis may offer greater diagnostic value, with acceleration and angular velocity data being as important as joint-angle measurements.

It is also worth noting that the sensor placed on the hip most frequently produced erroneous data records. This may have resulted from defects in that particular sensor or from its mounting location, which caused frequent interruptions in the line of sight between the sensor and the base station. Practical experience during the study also showed that sensors placed on the thighs often shifted or slipped, which may have further compromised the quality of the recorded data.

In the presented analysis, the LDA assumption of equal covariance matrices between classes was not always satisfied, which may have partially affected classification performance. As noted by Brobbey et al. [70], violating this assumption may reduce the accuracy of LDA, although it does not invalidate the usefulness of the resulting findings. Models that allow for variance differentiation, such as Quadratic Discriminant Analysis (QDA), often achieve higher effectiveness in such cases, but require larger datasets, which limits their use in exploratory analyses. In this study, the priority was not to construct an optimal classifier but to identify the features that best differentiate the studied groups; therefore, the obtained results retain significant cognitive value despite a partial violation of the LDA assumptions.

Importantly, even assessments performed by experienced physiotherapists are not fully repeatable. For instance, in a study on the reliability of the ARAT test in stroke survivors, the coefficient of concordance (Kendall’s W = 0.711) indicated good but incomplete agreement between specialists [71]. In the context of this study, a key limitation was that repetitions were assessed by a single evaluator, which restricts the objectivity of the results. Additionally, there were no clear guidelines on the positioning of the leading leg’s foot during the exercise, which may have contributed to variations in foot placement across both the correct and incorrect performance groups. This heterogeneity may explain the less frequent significance of ankle-related parameters among the angular features.

The analysis relied exclusively on simple statistical parameters and basic classification models, such as LDA and a single decision tree. For the decision tree, a block analysis was employed, grouping the features, which may have influenced how the model was trained. Alternative block-building strategies could yield different SHAP results and affect the interpretation of feature importance.

In summary, the presented results provide a valuable foundation for further experimental work. They highlight the potential of both angular and distance-based approaches and identify areas that warrant further investigation.

In subsequent research stages, it is advisable to apply more advanced machine learning methods, such as decision tree ensembles or neural networks, using the key biomechanical features identified in this study. Additionally, it is recommended to expand the exercise database, increase the size and diversity of the participant group, and develop a detailed movement assessment protocol covering all critical aspects of exercise performance. This approach will increase the stability of feature selection, improve classification accuracy, and better adapt the systems for use in both clinical and home rehabilitation settings.

## 5. Limitations and Future Work

This study, while identifying key biomechanical features, has several limitations. First, side-lunge performance was classified by a single physiotherapist, which limits objectivity and repeatability. Second, only 32 healthy participants were included (mean age 26.4 ± 8.5 years), limiting generalizability to clinical populations. General exercise guidelines lacked precise instructions for foot positioning, potentially increasing variability and affecting ankle-related parameters. Technical challenges included sensor shifts and intermittent line-of-sight loss, which may have affected data quality. Simple statistical parameters and highly interpretable models (LDA, single decision tree) were used, and SHAP feature-block grouping may have influenced feature importance assessments. Finally, VR goggles did not fully occlude vision, limiting ecological validity.

Anomalous samples, such as missing data, line-of-sight loss, or anatomically implausible spikes, were removed during preprocessing. No formal anomaly-detection criteria were established due to their sporadic and heterogeneous nature. Thus, the analysis focused on feature identification rather than model robustness under data-loss conditions. Future work will include explicit anomaly-detection criteria and sensitivity analyses.

The findings provide a foundation for further research. Future studies should include participants with a broader range of ages and motor abilities, especially clinical populations, and incorporate multiple independent evaluators to enable inter-rater agreement metrics. System performance should also be evaluated under non-professional, home-based conditions. While this study focused on interpretability, future work will examine more complex data-driven models (CNNs, LSTMs), validate the results against gold-standard motion analysis, and assess combined angular and distance features. Additionally, extending the analysis to other physical exercises will help generalise the findings across different movement patterns. Finally, a standardised protocol for movement execution, including foot orientation and stride range, will reduce variability and improve diagnostic relevance.

## 6. Conclusions

Angular and distance data provide complementary information: angular features yielded higher LDA classification effectiveness in distinguishing between correct and incorrect side lunge performances, whereas distance features proved more stable, resistant to calibration inaccuracies, and less computationally demanding, making them especially suitable for home applications.

Variables identified as significant were selected in both the global analysis (LDA) and the analysis of the local influence of individual parameters (SHAP). The high consistency of these approaches indicates the stability and reliability of the selected features.

The most important angular features included: hip joint flexion of the supporting limb, movement dynamics in this joint (angular acceleration of rotation and flexion/extension), and angular acceleration of rotation in the knee joint of the leading leg.

The key distance features comprised the distances between the goggles and the foot of the leading leg, between the shank of the leading leg and the shank of the supporting leg, and between the shank and foot of the leading leg, along with statistical measures (standard deviation, median) of the distances between the goggles and the thigh of the leading leg and between the shank and foot of the supporting leg.

An extensive set of sensors is not necessary for a reliable assessment of the side lunge. The analysis showed that the most useful information can be obtained from the goggles and sensors placed on the feet and shanks, while sensors on the hip and thighs are more prone to artefacts. As a result, it is possible to design simplified sensor configurations, appropriately adapted to home and clinical rehabilitation settings.

## Figures and Tables

**Table 1:** The final results of the angular data cleaning process.

Lower Limb	Joint	Number of Deleted Columns	% of All Samples
Stepping	hip	106	50.2%
	knee	58	27.5%
	ankle	36	17.1%
Stance	Hip	107	50.7%
	Knee	34	16.1%
	ankle	19	9%

**Table 2:** Sensor pairs and their assigned identification numbers.

Pair	Sensor 1	Sensor 2	Pair	Sensor 1	Sensor 2
1	Headset	Hip	15	Stepping limb thigh	Stepping limb foot
2		Stepping limb thigh	16		Stance limb thigh
3		Stepping limb shank	17		Stance limb shank
4		Stepping limb foot	18		Stance limb foot
5		Stance limb thigh	19	Stepping limb shank	Stepping limb foot
6		Stance limb shank	20		Stance limb thigh
7		Stance limb foot	21		Stance limb shank
8	Hip	Stepping limb thigh	22		Stance limb foot
9		Stepping limb shank	23	Stepping limb foot	Stance limb thigh
10		Stepping limb foot	24		Stance limb shank
11		Stance limb thigh	25		Stance limb foot
12		Stance limb shank	26	Stance limb thigh	Stance limb shank
13		Stance limb foot	27		Stance limb foot
14	Stepping limb thigh	Stepping limb shank	28	Stance limb shank	Stance limb foot

**Table 3:** Comparison of distance-based sensor sets: the three best overall, the two top-performing two-element and three-element sets, and, for illustration, the weakest three-element combination. Stability assessed using Std_F1 thresholds (<0.015 very stable; 0.015–0.025 acceptable; >0.025 increased variability).

Pair	Feature	Accuracy	F1	Std F1	AUC
Headset—Stance limb thigh	Std	0.74	0.72	0.154	0.79
Headset—Stance limb foot	Min				
Stepping limb foot—Stance limb shank	Median				
Stepping limb shank—Stepping limb foot	Std				
Headset—Stance limb foot	Medan	0.74	0.72	0.154	0.79
Stepping limb foot—Stance limb shank	Max				
Headset—Stance limb thigh	Q3				
Stepping limb shank—Stepping limb foot	Std				
Headset—Stance limb thigh	Std	0.74	0.72	0.154	0.78
Headset—Stance limb foot	Min				
Stepping limb foot—Stance limb shank	Std				
Stepping limb shank—Stance limb foot	Median				
Headset—Stance limb thigh	Std	0.71	0.71	0.142	0.74
Headset—Stance limb foot	Min				
Headset—Stance limb foot	Mean				
Headset—Stepping limb shank	Min	0.70	0.67	0.175	0.72
Headset—Stepping limb foot	Std				
Headset—Stance limb thigh	Std	0.55	0.50	0.20	0.59
Headset—Stance limb foot	Min				
Hip—Stepping limb thigh	Std				

**Table 4:** Best four-element set for angular data, with top two- and three-element sets included for comparison. Stability assessed using Std_F1 thresholds (<0.015 very stable; 0.015–0.025 acceptable variability; >0.025 increased variability). 1: Stepping limb; 2: Stance limb.

Kinematic Variable	Rotation	Feature	Accuracy	F1	Std F1	AUC
Angle	Hip Flexion/Extension (2)	Std	0.85	0.84	0.126	0.92
Angular Acceleration	Ankle Dorsi/Plantar (1)	Min				
	Knee Internal/External Rotation (2)	Q1				
angular Velocity	Hip Internal/External Rotation (2)	Median				
Angles	Hip Flexion/Extension (2)	Std	0.79	0.77	0.158	0.87
Angular Acceleration	Ankle Dorsi/Plantar (1)	Min				
Angle	Hip Flexion/Extension (2)	Std	0.85	0.83	0.018	0.89
Angular Acceleration	Ankle Dorsi/Plantar (1)	Min				
Angular Velocity	Hip External/Internal Rotation (2)	Median				

**Table 5:** Criteria used in a decision tree to select a set of features for subsequent stages of analysis.

Criterion	Limit Value	Description
Accuracy	≥0.68	First level of effectiveness: If the condition is met: all features from the block are included in further analysis.
F1	≥0.60	Second level of effectiveness: If Accuracy < 0.68 but mean F1 ≥ 0.60, the top 6 features (50%) are selected according to SHAP values.

**Table 6:** Results of metrics for sample blocks based on a decision tree with random validation applied to angular features.

Block Number (Angular Data)	Accuracy	Accuracy Std	F1	F1 Std
1	0.70	0.097	0.67	0.135
15	0.74	0.190	0.69	0.286
16	0.69	0.095	0.63	0.104
27	0.69	0.057	0.64	0.159

**Table 7:** Angular feature selection based on a decision tree with SHAP values (1: stepping limb, 2: stance limb).

Kinematic Variable	Rotation	Features
Angle	Hip Ab/Adduction (2)	ROM, Skew, Kurt
	Hip Internal/External Rotation (2)	Kurt, Skew
	Hip Flexion/Extension (2)	Std, ROM, Skew, Kurt
Angular Acceleration	Hip Flexion/Extension (1)	Std
	Knee Ab/Adduction (1)	Mean, Median, Q3
	Knee Internal/External Rotation (1)	Mean, Max, Min, Q1, Q3, Range, Std
	Knee Flexion/Extension (1)	Max, Mean, Median, Min, Q1, Q3, Range
Angular Velocity	Hip Flexion/Extension (1)	Std
	Knee Ab/Adduction (1)	Range, Q1, Q3, Std, Min, Mean
	Knee Internal/External Rotation (1)	Max, Mean

**Table 8:** Metric results for sample blocks based on a decision tree with random validation applied for distance features.

Number of Features	Accuracy	Accuracy Std	F1	F1 Std
82	0.72	0.077	0.73	0.087
41	0.77	0.069	0.77	0.070
21	0.77	0.075	0.78	0.078
11	0.80	0.075	0.80	0.082
6	0.78	0.062	0.78	0.069
2	0.73	0.063	0.73	0.073

**Table 9:** Feature selection results for joint-angle data using LDA and SHAP. 1: Stepping limb; 2: Stance limb.

Rotation	Feature	Number of Sets	% of Total	Selected by SHAP	|Effect Size|
Hip Flexion/Extension (2)	Std	468	10.6%	Yes	0.90
	ROM	292	6.6%	Yes	0.72
	Kurt	248	5.6%	Yes	0.42
Ankle Inv/Eversion (2)	Kurt	110	2.4%	No	0.23
Knee Flexion/Extension (2)	Skew	52	1.2%	No	0.39
Ankle Ab/Adduction (2)	Skew	51	1.2%	No	0.21
Ankle Inv/Eversion (2)	Std	47	1.6%	No	0.58
Ankle Dorsi/Plantar (2)	ROM	42	1%	No	0.49
Ankle Dorsi/Plantar (2)	Std	42	1%	No	0.54
Knee Internal/External Rotation (2)	ROM	40	0.9%	No	0.28
Ankle Inv/Eversion (2)	ROM	38	0.9%	No	0.52
Hip Ab/Adduction (1)	ROM	33	0.7%	No	0.40
Ankle Dorsi/Plantar (2)	Kurt	27	0.6%	No	0.23
Hip Flexion/Extension (1)	Std	6	0.1%	No	0.70
Hip Flexion/Extension (1)	ROM	3	0.07%	No	0.73

**Table 10:** Selected features combining joint angles, accelerations, and velocities using decision tree and SHAP analysis. 1: Stepping limb; 2: Stance limb.

Kinematic Variable	Feature	Rotation	Number of Sets	% of Total	Selected by SHAP	|Effect Size|
Angular Acceleration	Max	Knee Internal/External Rotation (1)	3136	71.1%	Yes	0.72
Angular Acceleration	Std		1014	23%	No	0.70
Angular Velocity	Min	Hip Internal/External Rotation (2)	577	13.1%	No	0.15
Angular Velocity	Min	Hip Flexion/Extension (2)	495	11.2%	No	0.26
Angle	Std	Hip Flexion/Extension (1)	468	10.6%	Yes	0.70
Angular Velocity	Median	Hip Internal/External Rotation (2)	314	7.1%	No	0.54
Angular Acceleration	Range	Hip Ab/Adduction (2)	313	7.1%	No	0.36
Angle	ROM	Hip Flexion/Extension (2)	292	6.6%	Yes	0.72
Angular Acceleration	Min	Hip Flexion/Extension (1)	272	6.2%	No	0.44
Angular Acceleration	Max	Hip Internal/External Rotation (2)	254	5.8%	No	0.18
Angle	Kurt	Hip Flexion/Extension (2)	248	5.6%	Yes	0.43
Angular Velocity	Range		215	4.9%	No	0.28
Angular Velocity	Std		208	4.7%	No	0.51
Angular Acceleration	Std		193	4.4%	No	0.24
Angular Acceleration	Q1	Hip Ab/Adduction (2)	181	4.1%	No	0.40
Angular Acceleration	Max		180	4.1%	No	0.55
Angular Velocity	Max	Hip Flexion/Extension (1)	180	4.1%	Yes	0.58
Angular Velocity	Max	Hip Flexion/Extension (2)	178	4%	No	0.27

**Table 11:** Selected distance-based features identified using decision tree and SHAP analysis.

Pair	Feature	Number of Sets	% of Total	Selected by SHAP	Effect Size
5	Std	36 (100%)	100%	No	0.60
7	Min	36 (100%)	100%	Yes	0.29
22	Mediana	21 (58.3%)	58.3%	No	0.64
19	Std	17 (47.2%)	47.2%	No	0.36
24	Median	7 (19.4%)	19.4%	No	0.63
7	Mean	7 (19.4%)	19.4%	No	0.34
21	Mean	4 (11.1%)	11.1%	Yes	0.63
19	Range	3 (8.3%)	8.3%	Yes	0.38
21	Max	3 (8.3%)	8.3%	No	0.59
22	Q3	2 (5.6%)	5.6%	No	0.59
24	Mean	2 (5.6%)	5.6%	No	0.59
25	Median	2 (5.6%)	5.6%	No	0.60
6	Min	2 (5.6%)	5.6%	No	0.33
6	Q1	2 (5.6%)	5.6%	No	0.40

**Table 12:** Importance of the selected biomechanical features.

Evaluation Criterion	Biomechanical Features of Correct Step Execution	The Significance of Selected Features
Starting position	Feet positioned parallel, hip-width apart	Shin to shin distance
	Upright trunk posture	Goggle–foot distance
	Neutral pelvis	Feature assignment is not applicable in this case.
Movement of the stepping limb	The limb moves sideways in a straight line, without rotation, diagonal shift, or forward displacement	Shin to shin distance
	The knee joint remains aligned above the ankle joint, without forward displacement. The hip is lowered by moving the pelvis backward, allowing controlled descent without overloading the knee joint	Distance between the shank of the stepping leg and its foot Knee joint rotation (acceleration) in stepping limb—should not be present.
Stance limb	Maintains extension or slight flexion in the knee joint	Combination of features: distance between the shank of the stepping leg and the supporting foot; distance between the shanks; distance between the goggles and the foot
	Foot remains stable, with no rotation or ground lift	It is worth paying attention to the range of motion in the ankle joint of the stance limb—the inversion/eversion of the foot. This feature did not appear in the SHAP analysis, but it was present in the effective LDA models
Torso and Pelvis	Trunk slightly inclined forward, maintaining the neutral curvature of the spine	The goggle–foot relation may indicate trunk inclination, but it does not reflect spinal curvatures. Angle in the hip joint of the stance limb
	No slouching—active postural control maintained	The goggle–foot relation may indirectly indicate slouching: when the posture is upright, this distance reaches its maximum value, whereas in the case of slouching, it decreases.
	Pelvis remains stable without rotation or inclination, maintaining symmetry	Feature assignment is not applicable in this case.
Range of Motion	Lowering of the centre of gravity occurs through simultaneous flexion of the hip and knee, without a forced large range of motion	The goggle–leading foot distance indirectly reflects the range of motion in these joints—when the movement is performed with a greater range, this distance decreases.
	Smooth, controlled movement within the physiological range	Change in the hip joint angle of the supporting limb, as well as acceleration in the knee joint and velocity in the hip joint of the stepping limb.
Return to the Starting Position	Push off from the stepping leg, return to the standing position	Angular velocity in the hip joint of the stepping limb Changes in shank-to-shank distance
	Maintained balance and trunk control.	Relations between goggles and other sensors.

## Data Availability

The original contributions presented in this study are included in the article. Further inquiries can be directed to the corresponding authors.

## Abbreviations

The following abbreviations are used in this manuscript:

LDA	Linear Discriminant Analysis
SHAP	SHapley Additive exPlanations
VR	Virtual Reality
HMQA	Human Motion Quality Assessment
FFT	Fast Fourier Transform
STFT	Short-Time Fourier Transform
DTW	Dynamic Time Warping
EMD	Empirical Mode Decomposition
HMM	Hidden Markov Model
GMM	Gaussian Mixture Model
ARMA	Autoregressive Moving Average
ARIMA	Autoregressive Integrated Moving Average
SVM	Support Vector Machine
KNN	k-Nearest Neighbours
PCA	Principal Component Analysis
ANN	Artificial Neural Network
LSTM	Long Short-Term Memory
BiLSTM	Bidirectional Long Short-Term Memory
CNN	Convolutional Neural Network
CNN LSTM	Convolutional Neural Network combined with LSTM
FCN	Fully Convolutional Network
ResNet	Residual Network
ROM	Range of Motion
AUC	Area under the curve
ROC	Receiver operating characteristic
